# Endovascular Management of Traumatic Aortic Arch Pseudoaneurysm With Concomitant Left Subclavian Artery Occlusion and Dissection: A Multidisciplinary Case Report

**DOI:** 10.7759/cureus.93182

**Published:** 2025-09-25

**Authors:** Sohaib Bassam Mahmoud Zoghoul, Saad Ur Rehman, Fajer Alishaq, Ahmad Zitoun, Amr Fares

**Affiliations:** 1 Interventional Radiology, Hamad Medical Corporation, Doha, QAT; 2 Vascular Surgery, Hamad General Hospital, Doha, QAT; 3 Vascular Surgery, Hamad Medical Corporation, Doha, QAT; 4 Vascular Surgery, Hamad Medical Corporation, DOHA, QAT

**Keywords:** blunt thoracic aortic injury, endovascular repair, hybrid intervention, pseudoaneurysm, subclavian artery occlusion, tevar, trauma

## Abstract

Blunt thoracic aortic injury (BTAI) with concurrent subclavian artery dissection poses complex challenges. This case highlights the role of multidisciplinary endovascular strategies in managing anatomically hostile injuries. We present a case of a 22-year-old male who presented with BTAI (grade 3 aortic arch pseudoaneurysm) and left subclavian artery (LSA) dissection/occlusion after a three-meter fall. Thoracic endovascular aortic repair (TEVAR) with a *Medtronic Valiant™ Captivia™ *system thoracic stent graft and LSA stenting using a *Fluency™ Plus* endovascular stent graft was performed. Postoperative imaging confirmed pseudoaneurysm exclusion and restored LSA flow. Multidisciplinary endovascular repair effectively addresses complex aortic and subclavian injuries. Ultra-short proximal landing zones (<2 mm) require precise stent deployment and hybrid revascularization. This case underscores the feasibility of endovascular repair in anatomically hostile trauma settings and highlights technical strategies that can guide management of similar complex injuries in young patients.

## Introduction

Blunt thoracic aortic injury (BTAI) carries high mortality without timely intervention [1]. Thoracic endovascular aortic repair (TEVAR) is now the preferred treatment, with lower morbidity than open surgery [2,3]. Grade 3 injuries, such as pseudoaneurysms, often require urgent repair, particularly when associated with mediastinal hematoma or subclavian artery involvement [4]. Subclavian artery injury occurs in up to 10% of BTAI cases and increases the risk of limb ischemia and vertebrobasilar insufficiency [5,6]. However, managing BTAI with ultra-short proximal landing zones and simultaneous subclavian artery repair in the trauma setting remains technically challenging and underreported. This case highlights a multidisciplinary endovascular approach addressing both issues in a young patient.

## Case presentation

A 22-year-old male sustained a three-meter fall onto his chest. On arrival, he was alert and oriented but in distress. Vital signs were as follows: temperature 36.7 °C, heart rate 72 bpm, respiratory rate 18/min, blood pressure 100/52 mmHg, and SpO₂ 100%. Examination revealed absent left brachial and radial pulses, with preserved pulses in the right upper and both lower extremities.

**Figure 1:**
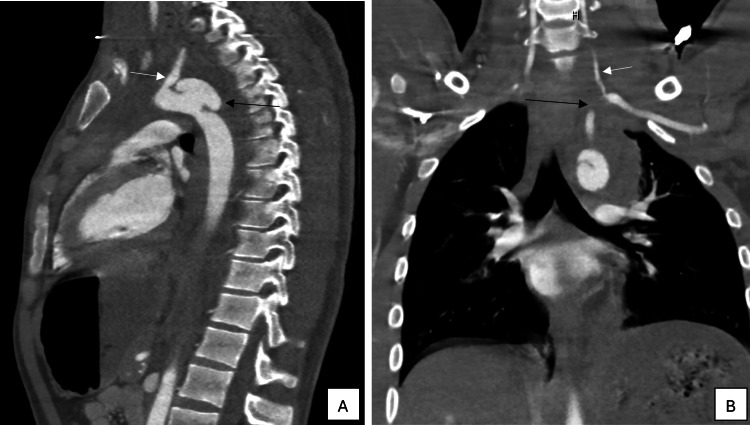
A: Saggital view CT angiogram of the thoracic aorta. B: Coronal view CT angiogram of the thorax. *A:* Large pseudoaneurysm (long black arrow) arising from the aortic arch juxtaductal to the left subclavian artery (short white arrow).* B:* Left proximal subclavian artery occlusion (long black arrow) with distal opacification by the left vertebral artery (short white arrow).

Initial imaging demonstrated a left clavicular fracture, first rib fracture, and mediastinal hematoma. CTA revealed a large juxtaductal aortic arch pseudoaneurysm (grade 3 BTAI) immediately adjacent to the left subclavian artery (LSA) origin, as well as a 4-cm LSA dissection with occlusion (Figure 1A-1B).

Given the ultra-short (<2 mm) proximal landing zone and concurrent LSA occlusion, a hybrid endovascular approach was chosen to ensure both exclusion of the pseudoaneurysm and restoration of subclavian perfusion:

TEVAR (vascular surgery)

**Figure 2:**
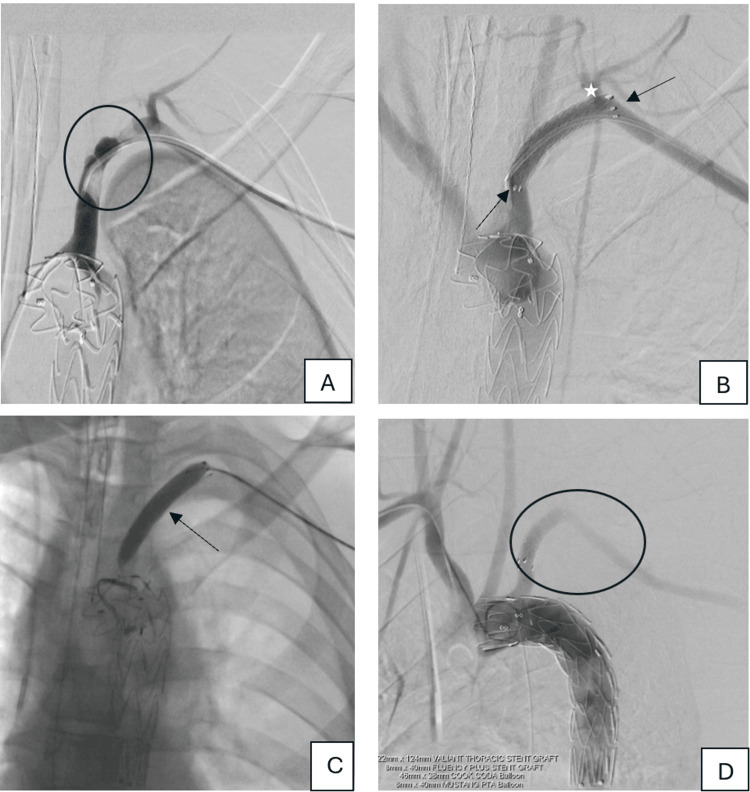
Digital subtraction angiography (DSA) of the aortic arch and thoracic aorta *A: *Digital subtraction angiography (DSA) through the right brachial access using PIG tail 5F angiographic catheter (Cordis, Hialeah, Florida, United States) showing the pseudoaneurysm (black arrow) juxta ductal to the left subclavian artery (white arrow).* B: *VALIANT thoracic stent graft 22 mm x 112 mm (black arrows) deployed successfully with the uncovered part of the stent covering the left subclavian artery origin, which shows normal proximal contrast opacification (white arrow).* C: *CODA balloon catheter (Cook, Bloomington, Indiana, United States) angioplasty was applied through the stent (white arrow).* D: *Final DSA shows exclusion of the pseudoaneurysm with normal opacification of the origin of left subclavian artery after the stent deployment (white arrow).

Through right femoral access, a Valiant™ Captivia™ thoracic stent graft (22 × 112 mm, Medtronic, Minneapolis, MN, USA) was deployed precisely distal to the LSA origin (Figure 2A-2D), achieving pseudoaneurysm exclusion while maintaining branch perfusion.

LSA stenting (interventional radiology)

**Figure 3:**
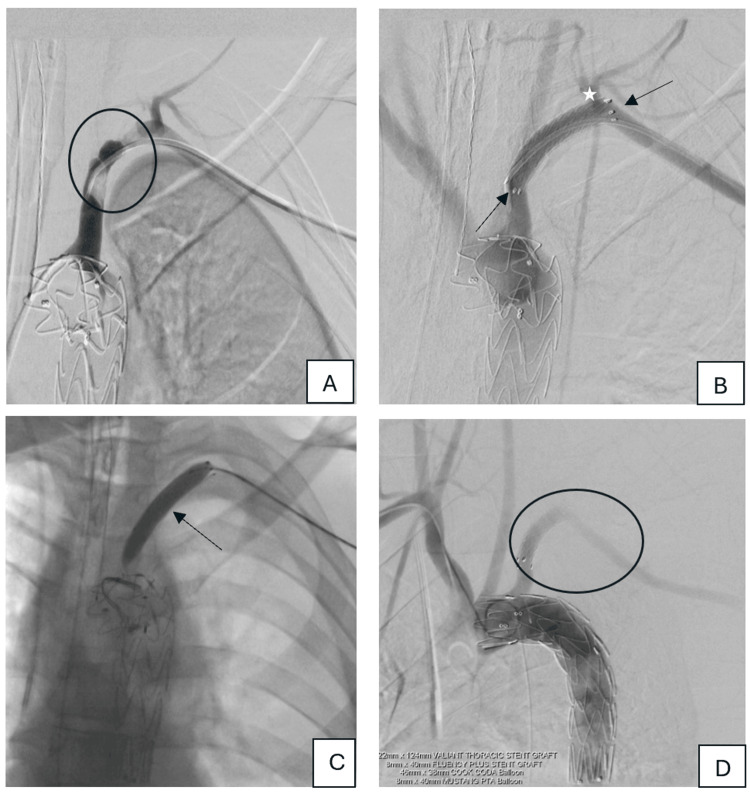
Digital subtraction angiography (DSA) of the left subclavian artery *A: *DSA shows abnormal contour and partial opacification of the left proximal subclavian artery (black circle) after IMPRESS® Berenstein angiographic catheter (MERIT, South Jordan, Utah, USA) used to cross the area of occlusion.* B: *Successful deployment of the FLUENCY PLUS stent graft 8 mm x 40 mm (black arrows) with an uncovered side part deployed at the normally opacified thyrocervical trunk (white star) and internal thoracic artery.* C: *MUSTANG 8 mm x 40 mm percutaneous transluminal balloon angioplasty was applied through the stent (black arrow).* D: *The final DSA shows satisfactory position of both stent grafts with restoration of the flow along the whole length of the left subclavian artery (black circle).

Via left brachial access, a Fluency™ Plus Endovascular Stent Graft (40 × 8 mm, BD, Franklin Lakes, NJ, USA) was placed across the dissected segment, followed by angioplasty with a Mustang™ balloon catheter (Boston Scientific, Marlborough, MA, USA), restoring antegrade LSA flow (Figure 3A-3D).

Postoperatively, the left radial pulse and triphasic Doppler signals were restored. CTA confirmed complete pseudoaneurysm exclusion and LSA patency. The patient was discharged on dual antiplatelet therapy, with a six-month follow-up showing sustained repair and no ischemia.

## Discussion

This case underscores key insights into managing complex BTAI:

Short landing zones

TEVAR in cases with ultra-short (<2 mm) proximal landing zones remains technically demanding, particularly in young trauma patients. Prior reports have described similar challenges, where precise deployment and adjunctive branch revascularization were required to achieve durable exclusion without compromising flow [7,8]. In our case, partial LSA coverage with subsequent stenting achieved seal and perfusion preservation, aligning with “revascularization-first” strategies. Balloon molding and meticulous intraoperative imaging are critical to minimize malposition or endoleak.

Concomitant subclavian repair

Concurrent LSA injury further complicates management. Covered stent grafts are increasingly employed in traumatic subclavian injuries, with several series reporting high technical success and one-year patency exceeding 90% [9]. While outcomes appear favorable compared to open repair, trauma data remain limited and heterogeneous; therefore, caution is warranted in generalizing superiority. Nonetheless, in this case, stenting provided rapid reperfusion and avoided the morbidity of open surgery.

Risks and complications

Despite the technical success, risks such as endoleak, stroke, or arm ischemia remain significant in TEVAR with arch involvement. Careful patient selection, perioperative neurologic monitoring, and surveillance imaging are essential to mitigate these complications.

Uncertainties and future directions

Although TEVAR demonstrates excellent acute efficacy, uncertainties persist regarding long-term device durability in young trauma patients with decades of life expectancy. Research is particularly needed on graft fatigue, reintervention rates, and optimal surveillance protocols. Annual CTA surveillance is recommended [10]. Future studies should explore standardized protocols for hybrid trauma interventions to optimize outcomes in this high-risk cohort. 

## Conclusions

This case demonstrates how a hybrid endovascular approach can restore both central aortic integrity and limb perfusion while avoiding the morbidity of open surgery in a young trauma patient. By combining TEVAR with subclavian stenting, hemorrhage was controlled and upper extremity ischemia was prevented in a single setting. The success of this strategy adds to the trauma literature by illustrating that multidisciplinary endovascular repair is feasible even in anatomically hostile situations, providing a valuable reference for managing simultaneous aortic and subclavian injuries.
